# Regulatory T Cell Responses to High-Dose Methylprednisolone in Active Systemic Lupus Erythematosus

**DOI:** 10.1371/journal.pone.0143689

**Published:** 2015-12-02

**Authors:** Alexis Mathian, Romain Jouenne, Driss Chader, Fleur Cohen-Aubart, Julien Haroche, Jehane Fadlallah, Laetitia Claër, Lucile Musset, Guy Gorochov, Zahir Amoura, Makoto Miyara

**Affiliations:** 1 Service de médecine interne 2, Centre de Référence National pour le Lupus et le Syndrome des Antiphospholipides, institut E3M, Groupement Hospitalier Pitié-Salpêtrière, Assistance Publique-Hôpitaux de Paris, Paris, France; 2 Sorbonne Universités, UPMC Univ Paris 06, Inserm UMRS1135, Centre d’Immunologie et des Maladies Infectieuses (Cimi-Paris), 83 Bd de l’hôpital, F-75013, Paris, France; 3 Département d’immunologie, Groupement Hospitalier Pitié-Salpêtrière, Assistance Publique-Hôpitaux de Paris, Paris, France; Instituto Nacional de Ciencias Medicas y Nutricion Salvador Zubiran, MEXICO

## Abstract

**Background/Purpose:**

A slight increase in the proportion of circulating regulatory T (Treg) cells has been reported in systemic lupus erythematosus (SLE) patients taking oral prednisone. The effects of intravenous (IV) high dose methylprednisolone (MP) on Tregs have not yet been described, especially in active SLE.

**Methods:**

We prospectively analyzed the proportion of circulating CD4^+^ Treg cell subsets defined as follows: (1) naïve Treg (nTreg) FoxP3^low^CD45RA^+^ cells; (2) effector Treg (eTreg) FoxP3^high^CD45RA^−^ cells; and (3) non-suppressive FoxP3^low^CD45RA^−^ cells (non-regulatory Foxp3^low^ T cells). Peripheral blood mononuclear cells of patients with active SLE were analyzed before the first infusion of IV high dose MP (day 0) and the following days (day 1, day 2, ±day 3 and ±day 8). The activity of SLE was assessed by the SLEDAI score.

**Results:**

Seventeen patients were included. Following MP infusions, the median (range) percentage of eTregs significantly increased from 1.62% (0.53–8.43) at day 0 to 2.80% (0.83–14.60) at day 1 (p = 0.003 versus day 0), 4.64% (0.50–12.40) at day 2 (p = 0.06 versus day 1) and 7.50% (1.02–20.70) at day 3 (p = 0.008 versus day 2), and declined to baseline values at day 8. Expanding eTreg cells were actively proliferating, as they expressed Ki-67. The frequency of non-regulatory FoxP3^low^ T cells decreased from 6.39% (3.20–17.70) at day 0 to 4.74% (1.03–9.72) at day 2 (p = 0.005); nTreg frequency did not change. All patients clinically improved immediately after MP pulses. The absence of flare after one year of follow up was associated with a higher frequency of eTregs at day 2.

**Conclusion:**

IV high dose MP induces a rapid, dramatic and transient increase in circulating regulatory T cells. This increase may participate in the preventive effect of MP on subsequent flares in SLE.

## Introduction

FoxP3-expressing regulatory T (Treg) cells are instrumental for the maintenance of self-tolerance. While the absence of Treg cells in scurfy mice and IPEX (Immune dysregulation, polyendocrinopathy, enteropathy, X-linked) patients bearing a dysfunctional FOXP3 gene leads to severe multisystemic lethal autoimmune disease [1–3], transfer of T cells devoid of Treg cells in nude mice leads to milder systemic autoimmunity, including gastritis, oophoritis and sometimes clinical and biological features resembling systemic lupus erythematosus (SLE), including arthritis, nephritis and the production of anti-double stranded DNA [4–6].

The seminal finding that a lack of Treg cells in adult mice could provoke a SLE-like disease in mice has led to numerous studies focused on Treg cell modifications in SLE.

Treg cells were first defined in humans as CD4^+^T cells harboring the alpha chain of the IL-2 receptor i.e., CD25 [7], following the seminal description by Sakaguchi *et al*. of Treg cells in mice [6]. The studies that have followed demonstrated that such Treg cells in humans also expressed the FoxP3 transcription factor [7]. However, subsequent studies have demonstrated that human FoxP3 expressing cells are heterogeneous in phenotype and function *i*.*e*., some circulating FoxP3 expressing cells are not suppressive [8, 9]. FoxP3^+^CD4^+^ regulatory T cells can be divided into CD4^+^CD45RA^−^FoxP3^bright^CD25^bright^ and CD4^+^CD45RA^+^FoxP3^+^CD25^+^ fractions, while the remaining CD4^+^FoxP3^+^ (i.e., CD4^+^CD45RA^−^FoxP3^low^CD25^+^) include a notable amount of non-regulatory, cytokine-secreting, activated T cells (= *non-regulatory FoxP3* ^*+*^ *T cells*). CD4^+^CD45RA^−^FoxP3^bright^CD25^bright^ Tregs are activated, highly suppressive and *in vivo* proliferating cells defined as *effector Tregs* (eTregs [8]) while CD4^+^CD45RA^+^FoxP3^+^CD25^+^ Tregs are fully functional and referred to as *naïve Tregs* (nTregs [8]). We have shown that the latter were highly increased during SLE flares, while effector Treg cells were decreased in most patients with SLE flares [8, 10]. These results are in line with numerous published reports showing an imbalance between Treg cells and effector T cells in active SLE [11, 12]. Numerous studies have also shown that the number of Treg cells returns to normal values when the disease is inactive [5, 10, 13]. Therefore, the manipulation of Treg cells to increase their number is considered an interesting potential therapeutic strategy to develop in SLE.

Administration of glucocorticoids is commonly used and has been proven efficient as a treatment for SLE flares irrespective of organ involvement [14, 15]. In severe flares, intravenous (IV) high dose methylprednisolone (MP) is useful to induce a rapid suppression of acute inflammation [16–19]. Hence, IV high dose MP pulses are recommended as part of the initial treatment regimen of severe lupus nephritis [20, 21] and can also be useful to obtain rapid beneficial effects on several types of non-renal lupus erythematosus [16–19]. While the broad actions of glucocorticoids on lymphocytes, neutrophils, mononuclear phagocytes and cytokines to induce anti-inflammatory and immunosuppressive effect are well known [19, 22, 23], their impact on Treg cells is less documented. Several studies have suggested that the induction of Treg cells may contribute to the immunosuppressive effects of glucocorticoids [24–28]. In SLE, a slight increase in the proportion of circulating Treg cells has been reported in patients taking oral prednisone [29–31]; the time to Treg cell recovery was reduced in patients treated with IV high dose MP [13]. However, to our knowledge, there has been no detailed report on the short-term effects of IV high dose MP on the different subsets of FoxP3^+^ T cells in active SLE until now.

Here, we show that IV high dose MP leads to a rapid, marked and transient increase in circulating effector Treg cells in most patients with active SLE. We also show that the expansion in effector Treg cells is associated with a better clinical outcome after one-year follow-up i.e., the absence of subsequent flares.

## Methods

### Patients

We conducted a prospective observational study between September 2011 and May 2013 in the department of internal medicine 2 (French national center for lupus and antiphospholipid syndrome) at Pitié Salpêtrière hospital, Paris, France. The inclusion criteria were to fulfill at least four of the 1997 American College of Rheumatology criteria for SLE [32] and to be treated for an SLE flare with high dose IV MP infusion (500 or 1000 mg repeated daily for 3 consecutive days, i.e., day 0, day 1 and day 2). At baseline, the clinical data and the Safety of Estrogens in Lupus Erythematosus National Assessment—Systemic Lupus Erythematosus Disease Activity Index (SELENA-SLEDAI) [33–35] were recorded. Routine measures were used to determine the anti-dsDNA antibodies titer (by Farr assay), anti-SSA/Ro, anti-SSB/La, anti-Sm, anti-RNP, anti-ribosome and anti-cardiolipin autoantibodies, the complement C3 level, complete blood count, urinary casts, proteinuria, pyuria and hematuria. Whenever required by the specific clinical situation, a complete blood count was also performed at day 2 which allowed us to determine the absolute count of lymphocytes at this date (pts # 1, 3, 4, 6, 8, 9, 11, 13, 16 and 17). After discharge from the hospital, all patients were closely monitored, initially on a monthly basis, for one year by a physician of the department of internal medicine 2. Clinical and biological data were systematically collected at 3, 6 and 12 months (month 3, month 6 and month 12) after MP pulses. Patients were asked to see their physician if a lupus flare occurred between the consultations. Lupus flares were defined according to the SELENA-SLEDAI Flair Index (SFI) [34, 35]. Bad responders were defined as patients having at least one lupus flare (mild, moderate or severe) during the one year follow-up after the high dose MP. The study was ethically approved by the CPP (Comités de Protection des Personnes) Ile de France VI and has been conducted according to the principles expressed in the Declaration of Helsinki. Patients provided written informed consent prior to their participation.

### Cell isolation and Flow Cytometry

Blood samples were collected into ACD (citric acid, citrate, dextrose) tubes before the first pulse of MP (baseline or day 0) and the following days until the patient’s discharge from the hospital. We were unable to collect blood samples at day 3 and day 8 for the patients discharged from the hospital before this time point. Whole peripheral blood mononuclear cells (PBMCs) were isolated through a Ficoll gradient (Eurobio, Les Ullis, France) and analyzed by flow cytometry (FACS Canto II, BD Bioscience). PBMCs were surface-stained with monoclonal antibodies: PerCP-conjugated-anti-CD4, APC-H7-conjugated-anti-CD45RA, BV450-conjugated-anti CD127, PeCy7-conjugated-anti CD25 (all from BD bioscience). Cells were then fixed and permeabilized using a fix/perm buffer (eBioSciences) following the manufacturer’s instructions and then intracellularly stained with PE-conjugated-anti-FoxP3 (259D clone) and FITC-conjugated-anti-Ki67 (BD Bioscience). The FoxP3 expressing CD4^+^ subset phenotype was defined as previously shown [8]. Naïve Treg cells were defined as CD4^+^CD45RA^+^FoxP3^low^ cells (nTreg) and effector Treg cells were defined as CD4^+^CD45RA^−^FoxP3^high^ cells (eTreg), while FoxP3 expressing non-regulatory Treg CD4^+^ T cells were defined as CD4^+^ CD45RA^−^FoxP3^low^ cells (non-reg FoxP3^+^ T cells).

### Statistical analysis

Values for quantitative variables were expressed as the median and range. Differences between groups were tested using the nonparametric Mann-Whitney U test and the Wilcoxon matched pairs signed ranks test. Statistical analyses were performed using GraphPad Prism, version 5.02 software (GraphPad Software, San Diego, CA, USA). All tests were 2-sided and a *p* value < 0.05 was considered statistically significant.

## Results

### Patient characteristics

Seventeen patients (pts) were included. Their baseline characteristics are described in Tables 1 and 2. All pts with a previously established SLE diagnosis were taking hydroxychloroquine (HCQ) except one. Eleven patients were treated with prednisone including nine with a dose of ≤ 10 mg/day. Immunosuppressive treatment was as follows: mycophenolate mofetil for 2 pts and methotrexate for 3 pts. All pts required hospitalization for SLE activity and were therefore considered to have a severe flare in the SELENA-SLEDAI Flare composite score [34, 35]. At inclusion, 12 pts had active arthritis and 7 had renal involvement (class III 3 pts, class V 2 pts, class IV 1 pt and class II 1 pt). Five pts developed a neuro-psychiatric involvement for which other causes than lupus were excluded (myelitis 2 pts, intracranial hypertension 1 pt, encephalitis 1 pt, sudden deafness 1 pt). Four pts had a pleurisy or a pericarditis. Patient #9 suffered from intracranial hypertension (nausea, vomiting, headache and blurred vision due to severe bilateral papilledema with raised intracranial pressure, aseptic meningitis and a normal MRI scan without cerebral venous sinus thrombosis), arthritis, positive Farr assay, positive Sm and low complement. The four pts (pts #1, 7, 12 and 16) for whom the musculoskeletal involvement was the main manifestation of the lupus flare suffered from a worse or new severe arthritis with an active synovitis ≥ 12 joints with marked loss of functional range of movements and significant impairment of activities of daily living, that has been present on several days. They all had to stop their professional activity due to the ongoing arthritis. Patients # 1, 7 and 16 received a lower dose of MP infusion, i.e., 500 mg per day for 3 consecutive days, while the fourteen other pts were treated with 1000 mg per day for 3 consecutive days. After the MP infusion, the baseline dose of prednisone was increased in 14 pts and a new immunosuppressant was introduced in 9 pts: cyclophosphamide for 4 pts, mycophenolate mofetil for 2 pts, azathioprine for 2 and belimumab for 1 patient (Table 2).

**Table 1:** Baseline characteristics and disease parameters of the SLE patients*.

	Patientsn = 17
Women	16 (94.1)
Age, median (range) years	34 (18–61)
Disease duration, median (range) years	5 (0–15)
*Previous organ involvement*	
Mucocutaneous or Musculoskeletal	12 (70.6)
Renal	2 (11.8)
Cardiorespiratory	2 (11.8)
Neuropsychiatric	2 (11.8)
Hematological	1 (5.9)
Daily prednisone	11 (64.7)
Hydroxychloroquine	13 (76.4)
Immunosuppressive agents^†^	5 (29.4)
No treatment	3 (17.7)
Positive Farr assay	13 (76.4)
Anti-SSa positive	8 (47.1)
Anti-SSb positive	3 (17.7)
Anti-Sm positive	6 (35.3)
Anti-RNP positive	7 (41.2)
Anti-ribosome positive	3 (17.7)
Anti-cardiolipin positive	8 (47.1)
Low C3	10 (58.8)
SELENA-SLEDAI score, median (range)	10 (2–26)

* except where indicated otherwise, values are the number (%) of patients. SLE = Systemic Lupus Erythematosus; SELENA-SLEDAI = Safety of Estrogens in Lupus Erythematosus National Assessment (SELENA) version of the SLE Disease Activity Index (SLEDAI).

^†^ Excluding antimalarial and prednisone.

**Table 2:** Baseline characteristics, disease parameters and treatment of the SLE patients.

#	Sex	Age (yrs)	Baseline characteristics (day 0)					Dose of methylprednisolone^†^ (mg)	Immediate treatment after the high dose of methylprednisolone		Treatment at month 12	
			Disease duration (yrs)	Daily Pred (mg)	Other treatments	Organ involvement	SLEDAI score*		Daily Pred (mg)	Other treatments	Daily Pred (mg)	Other treatments
**1**	F	56	4	10	MTX	Rash, arthritis	6	500	10	MTX, BMB	10	MTX
**2**	F	25	9	0	HCQ	GN (class IV), arthritis	24	1000	65	HCQ, MMF	10	HCQ, MMF
**3**	M	18	0	0	0	GN (class V), fever, rash, pleurisy, encephalitis	26	1000	55	HCQ, CYC	5	HCQ, MMF
**4**	F	29	7	5	HCQ	GN (class II), arthritis	14	1000	15	HCQ	5	HCQ
**5**	F	61	5	5	HCQ	Arthritis, myelitis	4	1000	65	HCQ, AZA	5	HCQ, MMF
**6**	F	54	8	5	HCQ, MMF	GN (class III), alopecia	14	1000	50	HCQ, CYC	20	HCQ
**7**	F	37	9	0	HCQ	Alopecia, arthritis	10	500	20	HCQ	10	HCQ
**8**	F	34	1	10	HCQ	GN (class III)	16	1000	30	HCQ, MMF	5	HCQ, MMF
**9**	F	33	0	0	0	Arthritis, aseptic, meningitis, intracranial hypertension	8	1000	60	HCQ	10	HCQ
**10**	F	23	1	30	HCQ	GN (class V), arthritis, alopecia	14	1000	30	HCQ	15	HCQ, MMF
**11**	F	30	2	20	HCQ, MMF	fever, arthritis, pericarditis	9	1000	20	HCQ	10	HCQ
**12**	F	38	6	9	HCQ, MTX	Arthritis	6	1000	20	HCQ, MTX	15	HCQ, MTX
**13**	F	52	2	5	HCQ, MTX	GN (class III), arthritis, alopecia, pericarditis	20	1000	70	HCQ, CYC	5	HCQ
**14**	F	39	13	5	HCQ	Pleurisy, arthritis	10	1000	20	HCQ	5	HCQ
**15**	F	42	0	0	0	Myelitis	2	1000	40	HCQ, CYC	5	HCQ, AZA
**16**	F	31	9	10	HCQ	mucosal ulcers, arthritis	8	500	15	HCQ, AZA	10	HCQ, MTX
**17**	F	28	15	0	HCQ	sudden deafness	10	1000	50	HCQ	6	HCQ

Pred = prednisone; MTX = methotrexate; BMB = belimumab; HCQ = hydroxychloroquine; MMF = mycophenolate mofetil; CYC = cyclophosphamide; AZA = azathioprine; GN = glomerulonephritis

* using the SELENA-SLEDAI [Safety of Estrogens in Lupus Erythematosus National Assessment (SELENA) version of the SLE Disease Activity Index (SLEDAI)].

^†^ the dose was delivered intravenously daily for three consecutive days.

### Effector Treg expansion following IV methylprednisolone pulses

**Fig 1:**
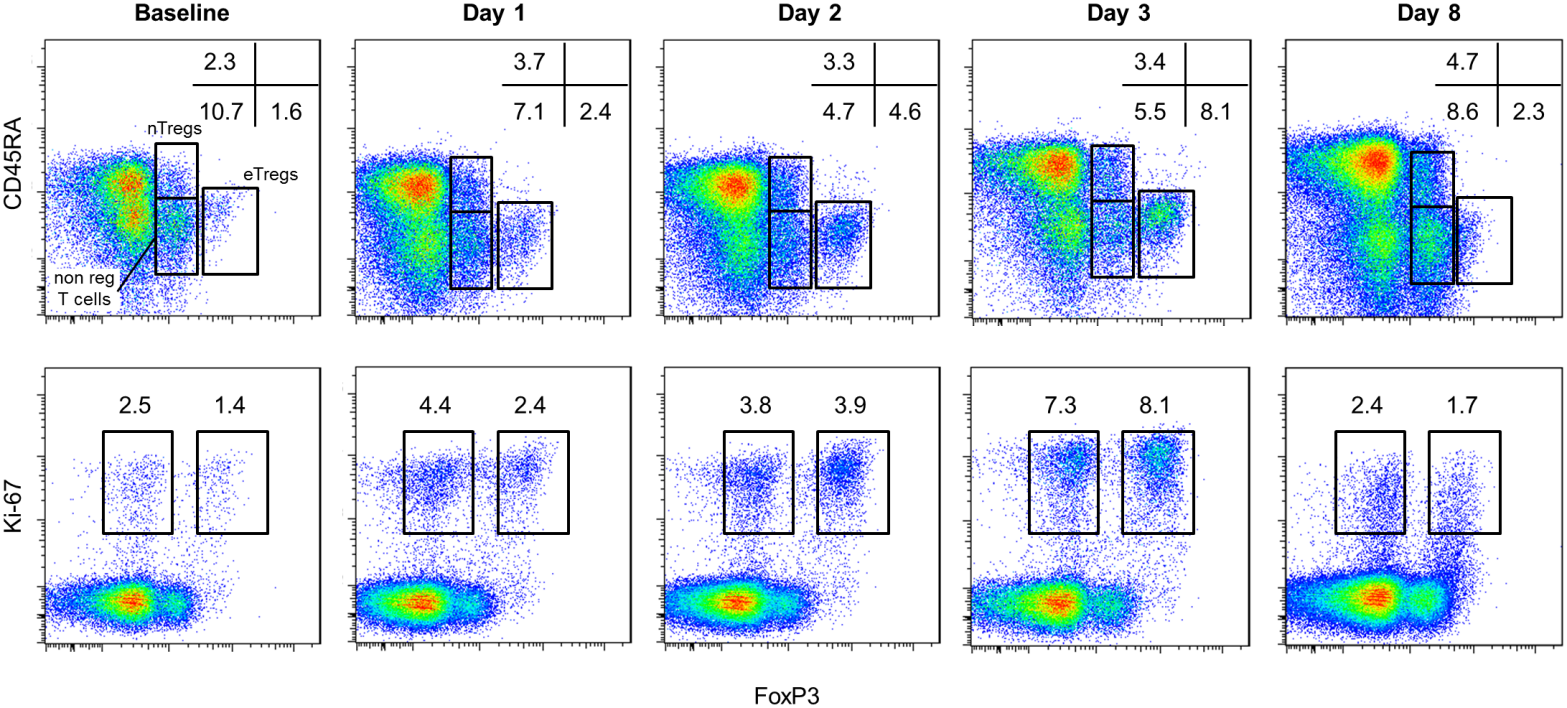
Cytofluorometric analysis of CD4^+^FoxP3^+^ T cell subsets following IV methylprednisolone pulses in SLE patients. Fresh PBMCs from SLE patients were analyzed by flow cytofluorometry, gated on CD4^+^ T lymphocytes, for the expression of FoxP3 and CD45RA (top rows) and Ki-67 (bottom rows). FoxP3^+^CD4^+^ T cells can be divided into CD4^+^CD45RA^−^FoxP3^bright^ effector Tregs (eTregs) and CD4^+^CD45RA^+^FoxP3^+^ naïve Tregs (nTregs), while the remaining CD4^+^CD45RA^−^FoxP3^low^ include a notable amount of non-regulatory, cytokine-secreting, activated T cells (non-regulatory FoxP3^+^ T cells) [8]. FoxP3^high^Ki-67^+^ (right gate), which correspond to the eTregs, and FoxP3^−^Ki-67^+^ (left gate), which correspond to non-regulatory T-cells, are shown on the lower FACS panel. Percentages of the different subsets are shown. Representative analyses from one SLE patient are shown (pt #3).

At baseline, in accordance with our published works [8, 10], the median (range) percentages of CD45RA^−^FoxP3^high^ eTreg, CD45RA^+^FoxP3^low^ nTreg and non-regulatory FoxP3^low^ T cells were 1.62% (0.53–8.43), 2.48% (0.87–5.58) and 6.39% (3.20–17.70), respectively (Table 3 and Fig 1).

**Table 3:** Evolution of FoxP3^+^ T cell subsets and disease activity in SLE patients following high dose methylprednisolone.

#	effector Tregs				naïve Tregs			non-reg Foxp3^+^ T cells			SLEDAI score*			No. of flaresat month 12
	day 0^†^	day 2	day 3	fold increase^‡^	day 0	day 2	day 3	day 0	day 2	day 3	month 3	month 6	month 12	
**1**	1.43	6.22		4.35	2.76	0.63		5.22	3.90		0	0	0	1 mild or moderate (Rash at M7)
**2**	1.33	3.63	9.55	2.73	1.78	0.80	1.64	6.39	4.14	3.15	6	2	2	0
**3**	1.62	4.64	8.09	2.86	2.29	3.30	3.35	10.72	4.74	5.53	4	4	2	0
**4**	1.35	6.83		5.06	3.99	4.26		6.61	6.94		2	2	2	0
**5**	0.97	3.49	4.23	3.60	1.41	5.77	3.33	3.69	2.91	2.71	0	0	0	1 severe (Myelitis at M3)
**6**	1.64	7.97	9.24	4.86	5.58	1.85	0.64	7.70	4.83	3.65	10	10	6	0
**7**	8.43	12.40	20.70	1.47	0.94	0.83	0.74	16.50	6.97	9.00	4	4	4	0
**8**	0.53	1.07		2.02	2.86	3.73		3.20	2.62		12	6	2	0
**9**	5.80	6.04	6.90	1.04	4.50	2.73	1.90	17.70	5.40	5.40	2	2	2	0
**10**	0.92	0.50	1.02	0.54	3.32	1.65	1.63	6.34	1.03	1.85	4	6	0	3 severe (Arthritis at M1, M6 and M9)
**11**	1.90	2.66		1.40	3.87	0.53		8.07	4.03		2	2	2	1 mild or moderate (Arthritis at M11)
**12**	1.31	2.10		1.60	0.87	0.43		4.84	3.76		6	6	6	5 mild or moderate (Arthritis at M3, M6, M8, M9 and M10)
**13**	2.59	3.64	3.84	1.41	2.71	6.78	2.77	10.58	9.10	8.46	10	2	2	0
**14**	1.64	6.30		3.84	0.93	0.69		14.07	6.60		4	4	4	0
**15**	4.25	6.74		1.59	2.45	2.40		5.70	9.72		0	0	0	0
**16**	1.84	2.87		1.56	2.48	2.90		5.14	6.13		4	8	8	1 severe (arthritis at M6) 1 mild to moderate (arthritis at M12)
**17**	1.49	6.94		4.66	1.03	0.50		6.39	4.66		2	2	2	0

* using the SELENA-SLEDAI [Safety of Estrogens in Lupus Erythematosus National Assessment (SELENA) version of the SLE Disease Activity Index (SLEDAI)].

^†^ values are the percentage of the subset among CD4^+^ T lymphocytes.

^‡^ values are the ratio of effector Tregs between day 2 and day 0.

^¶^ flares occurring during the 12 months which followed the high dose methylprednisolone were defined according to the SELENA-SLEDAI Flair Index (SFI).

**Fig 2:**
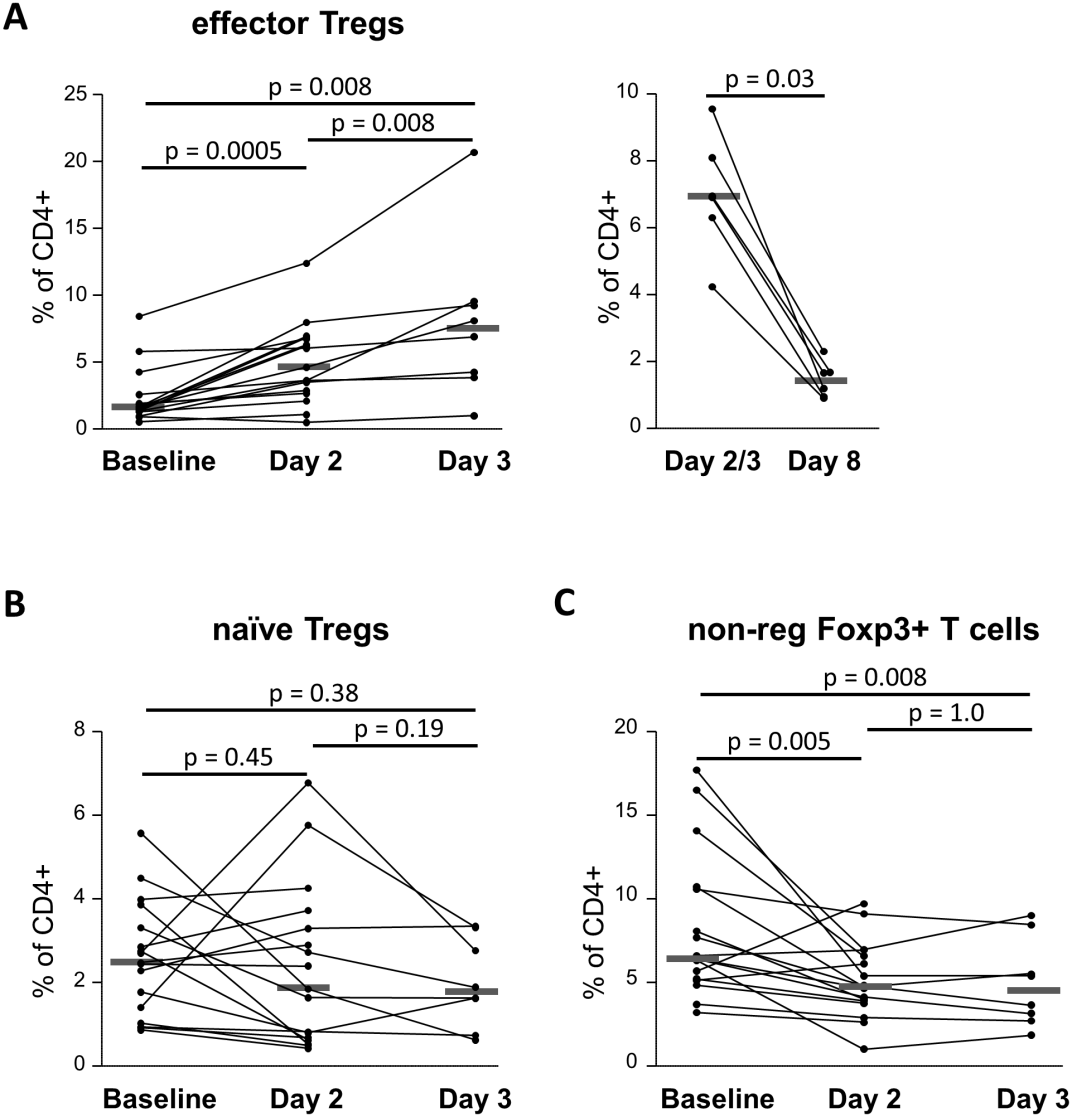
Frequencies of CD4^+^FoxP3^+^ T cell subsets following IV methylprednisolone pulses in SLE patients. Fresh PBMCs from SLE patients were analyzed by flow cytofluorometry as described in Fig 1. Kinetics of **(A)** effector Tregs, **(B)** naïve Tregs and **(C)** non-regulatory FoxP3^+^ T cells were performed in patients with active SLE undergoing IV high dose MP pulse treatment at baseline. Seventeen patients at baseline and day 2, 8 patients at day 3 and 6 patients at day 8 were assessed. (A) Right panel, eTreg cells frequencies at day 3 are displayed (except for two patients for whom these data are missing; eTreg cells frequencies at day 2 are shown instead). Each dot represents an individual assessed in an independent experiment, and the grey bar shows median values. Statistical analyses were performed using the Wilcoxon matched pairs signed ranks test.

Following MP therapy, we observed a marked increase in the eTregs subset in all patients except pt #10 (Table 3 and Figs 1 and 2A). One representative patient FoxP3^+^CD4^+^ T cells subset kinetics is shown (Fig 1). The median percentage of CD45RA^−^FoxP3^high^ eTregs significantly increased at day 1 at 2.80% (0.83–14.60) (p = 0.003 versus day 0) (data not shown) and even more at day 2 [4.64% (0.50–12.40), p = 0.0005 versus day 0 and p = 0.06 versus day 1] as well as at day 3 [7.50% (1.02–20.70), p = 0.008 versus day 0 and p = 0.008 versus day 2)] (Table 3, Fig 2A and data not shown,). The increase in the proportion of eTregs among CD4 T cells that we observed at day 2 corresponds to a significant increase in the count of eTreg cells (S1 Fig). The range of the expansion was variable. While most patients doubled their proportion of eTreg cells from baseline values at day 2 (Table 3), we observed that some patients displayed an increase in eTreg cells superior to a 5-fold at either D2 or D3 (e.g., patient #4 at day 2 and patients #2, 3 and 6 at day 3) and other patients showed an increase below 1.6 (pts #7, 9, 11, 12, 13, 15 and 16). Thus, these results indicate that high dose IV MP induces a variable but significant increase in circulating eTreg cells.

When analyzing the phenotype of eTreg cells at day 2 and day 3, we observed that most eTreg cells expressed the intranuclear proliferation marker Ki-67, indicating that MP may ultimately result in increased eTreg cell proliferation (Fig 1). Approximately 90% of eTregs cells at day 2 expressed CD15s, a marker that is highly specific for activated, terminally differentiated, and suppressive eTreg cells (S2 Fig) [36]. In pts treated with MP, only a small proportion of eTregs expressed CD45RA (Fig 1) and only a very small subset of CD45RA^+^ CD4^+^ T cells were Ki-67^+^ (data not shown). Effector Tregs did not express Annexin V (data not shown). Because we also observed a small decrease in nTreg cells, although not statistically significant (Fig 2B), we cannot exclude a concomitant enhanced conversion of nTreg cells to eTreg cells.

Finally, the expansion of eTregs was transient, as their proportion declined to baseline values by day 8 following the first pulse of MP in all of the 6 patients who were evaluated (Fig 2A). For these 6 patients, the median frequencies of eTreg cells increased from 1.56% (0.97–5.80) at day 0 to 6.92% (4.23–9.55) at day 3 (for the two patients for whom the day 3 data are missing, the eTreg cells frequencies at day 2 were taken instead) (p = 0.031 versus day 0) only to decrease to 1.43% (0.90–2.30) at day 8 (p = 0.031 versus day 2/3 and p = 0.60 versus day 0) (Fig 2A).

### Non-regulatory FoxP3^+^ T cells markedly decrease following IV methylprednisolone pulses

A significant decrease in the median frequency of non-regulatory CD45RA^−^FoxP3^low^ T cells was observed from 6.39% (3.20–17.70) at day 0 to 4.74% (1.03–9.72) at day 2, p = 0.005, and 4.53% (1.85–9.00) at day 3, p = 0.008 (Table 3 and Fig 2C). Non-regulatory FoxP3 T cells did not express Annexin V (data not shown).

### Disease improvement after methylprednisolone pulse

**Fig 3:**
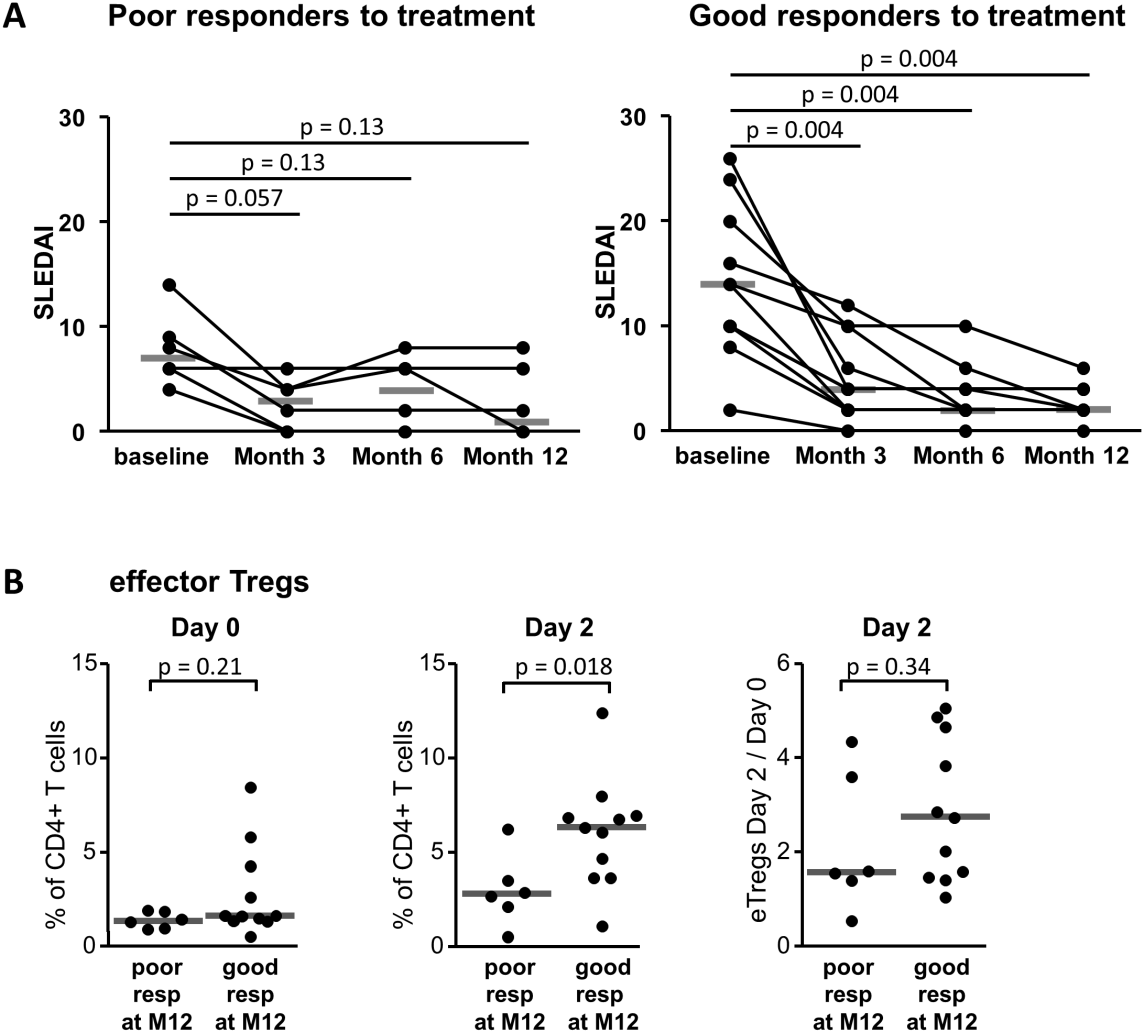
Frequencies of eTreg cells in SLE patients according to their clinical response following IV methylprednisolone pulses. **(A)** Evolution of the SELENA-SLEDAI score at baseline, month 3, 6 and 12 following IV MP pulses according to the responder status of the patients defined after 12 months of follow-up. **(B)** Effector Treg frequencies at day 0 (baseline) and day 2 and the ratio of effector Tregs between day 2 and day 0 in poor and good responders after 12 months of follow-up. (A-B) Each dot represents an individual and lines show median values. Statistical analyses were performed using the Wilcoxon matched pairs signed ranks test (A) and the Mann-Whitney *U* test (B).

Following the IV MP infusions and the subsequent changes in the daily treatment, all patients initially improved (Table 3, Fig 3A and data not shown). At month 3, the SLEDAI score improved compared to baseline for all pts except patient #12. The median SLEDAI score decrease from 10 (2–26) at day 0 to 4 (0–12) at month 3, p = 0.0005. However, this beneficial effect was not maintained for all patients: 3 pts experienced an early relapse (pts #5, 10 and 12) and, after one year of follow-up, 3 more pts had experienced at least one new lupus flare (pts #1, 11 and 16) (Table 3). Thus, six patients were classified as bad responders one year after MP pulse therapy. The evolution of the SLEDAI score according to the responder status is displayed in Fig 3. Even the patients classified as bad responders showed a slight, but not significant, improvement in disease activity (Fig 3A).

### eTreg expansion is associated with a better clinical outcome

Several lines of evidence have shown that regulatory T cells can prevent the occurrence of autoimmune responses. As a result, the use of Treg cell expansion has become an attractive target for the prevention of autoimmune disorders [37]. We therefore investigated whether the expansion of eTreg cells following IV MP pulses was associated with the absence of subsequent flares. The proportions of eTreg cells among CD4^+^ T cells two days after IV MP pulses were higher in the patients with a good clinical response during the year which followed the MP pulses (Fig 3B). We also compared the fold expansion of eTreg cells at day 2 between patients with good responses and those with a poor outcome at month 12 and found no significant difference (Fig 3B).

## Discussion

It is known that glucocorticoids, commonly used to treat SLE, exert a broad range of effects on the immune system. As some studies have shown that oral steroid drugs could also influence the regulatory cell population, we postulated that IV high dose MP could have a dramatic effect on Treg cells. In the present study, we show that exposure to IV high dose MP pulses induced a marked eTreg increase and a rapid clinical improvement in almost all SLE patients studied. We also showed that a good clinical outcome after one year of follow up was associated with a higher frequency of eTregs at day 2. It can therefore be envisaged that the expansion of eTregs plays a role in the anti-inflammatory and immunosuppressive effect of glucocorticoids, especially for the high concentrations achieved by parenteral administration.

The influence of glucocorticoid therapy on regulatory T cells is still a matter of debate. Several studies have shown that glucocorticoid drugs increase *in vitro* and *in vivo* FoxP3 expression and could induce the expansion of CD4^+^CD25^+^FoxP3^+^ regulatory T cells *in vivo* [13, 24–31, 38]. These conclusions have been challenged by two other studies [39, 40]. Sbiera *et al*. showed that short-term glucocorticoids have little impact on the relative frequency of circulating Treg cells in patients without an inflammatory disease and in mice receiving high-dose IV prednisolone [39]. In this study, Tregs were monitored 14 days after treatment. Here, on the contrary, we clearly showed a dramatic expansion of peripheral Treg cells after IV high dose MP in active SLE. The fact that this expansion was early (i.e., seen as soon as 24 hours after the first pulse) and transient (i.e., not present after 8 days) certainly explains the discrepancy between the two studies. It is also possible that the steroid effect observed on Tregs is disease-dependent, as CD4^+^ T cell homeostasis is markedly perturbed in Lupus patients. Indeed, we did not observe such dramatic eTreg expansions in the steroid-treated patients suffering from other autoimmune diseases that we could study (Sarcoidosis (n = 6), vasculitis (n = 2), myositis (n = 2), data not shown). In transplanted patients treated with MP for acute kidney rejection, Seissler *et al*. observed an increase in a specific subset of Treg cells that displayed the CD4^+^FoxP3^+^CD127^low^CD45RA^−^HLA-DR^+^ phenotype [40], but they did not observe a global Treg increase. The reported results may be explained by the concomitant use of several immunosuppressors such as cyclosporine A that might have compromised Treg cell proliferation [41, 42]. As the increase in the proportion of eTregs among CD4 T cells corresponds to a significant increase in the eTreg counts, we can exclude that the over-representation of eTregs among CD4^+^ T cells would be solely explained by the selective cell death of CD25^−^FoxP3^−^ CD4^+^ T cells [43]. Steroid-induced apoptosis [22] is nevertheless an important mechanism that should be taken into account. Indeed, the non-regulatory Foxp3^low^ T cell decrease we observed could be related to apoptosis. However, this interpretation remains speculative, as we could not detect Annexin V^+^ non-regulatory Foxp3^low^ T cells, possibly due to their rapid clearance from the circulation (data not shown).

The effects of steroids are dependent on their binding to the cytoplasmic glucocorticoid receptor (GCR) and their translocation to the nucleus. In the nucleus, the steroid-GCR complex acts as a transcription factor that enhances the expression of numerous genes [44]. Because GCR has been described as an enhancer for FoxP3 gene expression [45], it is highly likely that the eTreg cell expansion that follows the MP pulse in SLE is the consequence of the enhancement of FoxP3 gene expression. However, whether the increase in eTregs is a result of higher FoxP3 expression that would drive FoxP3^−^ and FoxP3^low^ cells to FoxP3^high^ or is a consequence of the proliferation of pre-existing eTreg cells cannot be concluded from the present data. Experiments in mice tracking these different subsets and analysis of the T-cell receptor repertoire of regulatory T cells could help resolve this question.

Because Treg cells are instrumental for the maintenance of self-tolerance, several groups have attempted to manipulate Treg cells *in vitro* or *in vivo* to treat autoimmune diseases [37]. In this regard, Treg transfer therapy is expected to be an efficacious way to prevent organ rejection, graft versus host disease or autoimmune disorders. Different animal models have indicated that the infusion of Treg cells in SLE prone mice was efficient in preventing the development of diseases [46, 47]. In the (NZB×NZW)F1 lupus prone mouse, the progression of nephritis was delayed by the transfer of Tregs from young F1 mice. As Treg transfer therapy has not yet proven its efficiency in human SLE, an alternative strategy is to increase Treg cells *in vivo*. We have shown here that high dose IV MP *per se* is sufficient for the expansion of eTreg cells. High dose IV methylprednisolone is still of value in treating patients with SLE whose disease is not controlled. It provides symptomatic relief and the regression of severe forms of disease and may also enable a reduction in the dose of maintenance glucocorticoids [18, 19]. The high concentrations of glucocorticoids achieved by IV pulse appears to have a more immediate and profound effect on Treg cells compared to conventional oral treatment [48]. However, despite the clinical efficacy of pulse for the initial management of active SLE, this benefit is not maintained for prolonged periods [17, 49]. This has to be put into perspective with the observation that eTreg cell expansion is only transient. Because repeated use of high dose IV MP is limited by the increased risk of infection [18, 19, 50], alternative strategies have to be developed to sustain Treg cell expansion over the long term. One of these strategies is to increase Treg cells *in vivo* using Treg cell related cytokines such as IL-2. Three phase I/II trials have evaluated the effects of IL-2 in chronic GVH, type 1 diabetes and HCV related infectious cryoglobulinemic vasculitis [51–53]. While IL-2 was shown to significantly increase the proportion of circulating Treg cells in all studies, this cytokine was also shown to ameliorate skin involvement in GVHD as well as in vasculitis, but was inefficient in type 1 diabetes.

The use of IL-2 in SLE seems particularly interesting because a reduction of this cytokine induced an imbalance of Treg cells and effector T cells leading to accelerated disease progression in the lupus prone mouse [54]. Administration of IL-2 promotes the homeostatic proliferation of endogenous Tregs, delays progression of the disease [54, 55] and corrects regulatory T cell defects in patients SLE [56]. A case of a patient with refractory SLE successfully treated with IL-2 has been reported recently [57]. Due to their synergistic effect, the combination of glucocorticoids and IL-2 also seems particularly interesting [58].

Our results should nevertheless be interpreted with a few limitations in mind. It is important to underline that the clinical outcome depends also on the maintenance of remission and probably not only on a transient increase in Tregs. Furthermore, one cannot discern with certainty whether all Treg cells need to expand to elicit a clinical response. It could be possible that the expansion of specific clones is the determining factor to control autoimmunity. In addition, a change in Tregs in the peripheral blood cannot predict the action of Treg cells at the site of inflammation and the level of regulation that they exert there. It was also demonstrated by others that the increase in Treg cells associated with SLE remission seems to be independent of the treatment used [13]. However, the increase in effector Treg cells that we observed shortly after the MP pulses is more in favor of a direct impact of glucocorticosteroids on the regulatory T cell physiology rather than a consequence of disease remission and/or the concomitant use of other immunosuppressive drugs. We showed that expanding eTreg cells upon high dose IV MP led to CD15s expression, a marker highly specific for activated, terminally differentiated, and mostly suppressive eTreg cells which enabled us to conclude that IV MP induces bona fide regulatory T cells [36]. However, we did not study other known markers for Tregs such as CD127 [59] and Helios [60, 61] which have already been evaluated in lupus [62–65] or new markers such as TIGIT [66] or FCRL3 [67]. It might be worthwhile to include these markers in further studies to better characterize these cells.

Altogether, we showed that high dose IV MP leads to a rapid, marked and transient increase in circulating effector Treg cells in most patients with active SLE. This was associated with a clinical improvement immediately after the MP pulses. Importantly, most of the patients with a better Treg expansion did not experience new flares after 1 year of follow-up. This indicates that high dose IV MP may prevent flares by increasing the number of eTreg cells. We therefore suggest that therapeutic strategies aimed at maintaining expanding Treg cells over the long term may help to prolong the beneficial effect of MP IV pulses. Our results also strengthen the rationale for the therapeutic use of Treg cells in the prevention of flares in autoimmune diseases with a remitting-relapsing course. Due to the limited sample size of our study group, additional evidence should be gathered in SLE patients.

## Supporting Information

S1 Fig Numbers of circulating effector Tregs before and after IV high dose methylprednisolone in SLE patients. The frequency of eTreg were determined by flow cytofluorometry as shown in Fig 1. The absolute counts for circulating eTreg cells were calculated by multiplying the frequencies of eTreg cells among CD4^+^ T cells with the frequencies of CD4^+^ T cells among total lymphocytes and the total lymphocyte blood count which was available at day 3 only for 10 patients. Each dot represents an individual assessed in an independent experiment, and the grey bar shows median values. Statistical analyses were performed using the Wilcoxon matched pairs signed ranks test.
(TIF)

S2 Fig CD15s expression in CD4^+^FoxP3^+^ T cell subsets following IV high dose methylprednisolone pulses in SLE patients. Fresh PBMCs from SLE patients were analyzed by flow cytofluorometry, gated on CD4^+^ T lymphocytes, for the expression of FoxP3 and CD15s before IV high dose MP pulses i.e., day 0 and at day 2 after the first pulse. Percentages of the different subsets are shown. Representative analyses from one SLE patient are shown (pt #15).
(TIF)

## References

[1] Khattri R, Cox T, Yasayko SA, Ramsdell F. An essential role for Scurfin in CD4+CD25+ T regulatory cells. Nat Immunol. 2003;4(4):337–42. Epub 2003/03/04. 10.1038/ni909 . 12612581

[2] Fontenot JD, Gavin MA, Rudensky AY. Foxp3 programs the development and function of CD4+CD25+ regulatory T cells. Nat Immunol. 2003;4(4):330–6. Epub 2003/03/04. 10.1038/ni904 . 12612578

[3] Hori S, Nomura T, Sakaguchi S. Control of regulatory T cell development by the transcription factor Foxp3. Science. 2003;299(5609):1057–61. Epub 2003/01/11. 10.1126/science.1079490 . 12522256

[4] Sakaguchi S, Yamaguchi T, Nomura T, Ono M. Regulatory T cells and immune tolerance. Cell. 2008;133(5):775–87. Epub 2008/05/31. 10.1016/j.cell.2008.05.009 S0092-8674(08)00624-7 [pii]. . 18510923

[5] Miyara M, Gorochov G, Ehrenstein M, Musset L, Sakaguchi S, Amoura Z. Human FoxP3+ regulatory T cells in systemic autoimmune diseases. Autoimmun Rev. 2011;10(12):744–55. Epub 2011/05/31. 10.1016/j.autrev.2011.05.004 S1568-9972(11)00106-6 [pii]. . 21621000

[6] Sakaguchi S, Sakaguchi N, Asano M, Itoh M, Toda M. Immunologic self-tolerance maintained by activated T cells expressing IL-2 receptor alpha-chains (CD25). Breakdown of a single mechanism of self-tolerance causes various autoimmune diseases. J Immunol. 1995;155(3):1151–64. Epub 1995/08/01. . 7636184

[7] Sakaguchi S, Miyara M, Costantino CM, Hafler DA. FOXP3+ regulatory T cells in the human immune system. Nat Rev Immunol. 2010;10(7):490–500. Epub 2010/06/19. 10.1038/nri2785 nri2785 [pii]. . 20559327

[8] Miyara M, Yoshioka Y, Kitoh A, Shima T, Wing K, Niwa A, et al Functional delineation and differentiation dynamics of human CD4+ T cells expressing the FoxP3 transcription factor. Immunity. 2009;30(6):899–911. Epub 2009/05/26. 10.1016/j.immuni.2009.03.019 S1074-7613(09)00202-7 [pii]. . 19464196

[9] d'Hennezel E, Yurchenko E, Sgouroudis E, Hay V, Piccirillo CA. Single-cell analysis of the human T regulatory population uncovers functional heterogeneity and instability within FOXP3+ cells. J Immunol. 2011;186(12):6788–97. Epub 2011/05/18. 10.4049/jimmunol.1100269 jimmunol.1100269 [pii]. . 21576508

[10] Miyara M, Amoura Z, Parizot C, Badoual C, Dorgham K, Trad S, et al Global natural regulatory T cell depletion in active systemic lupus erythematosus. J Immunol. 2005;175(12):8392–400. Epub 2005/12/13. doi: 175/12/8392 [pii]. . 16339581 10.4049/jimmunol.175.12.8392

[11] Crispin JC, Martinez A, Alcocer-Varela J. Quantification of regulatory T cells in patients with systemic lupus erythematosus. Journal of autoimmunity. 2003;21(3):273–6. Epub 2003/11/06. . 14599852 10.1016/s0896-8411(03)00121-5

[12] Mellor-Pita S, Citores MJ, Castejon R, Tutor-Ureta P, Yebra-Bango M, Andreu JL, et al Decrease of regulatory T cells in patients with systemic lupus erythematosus. Ann Rheum Dis. 2006;65(4):553–4. Epub 2006/03/15. 10.1136/ard.2005.044974 16531555 PMC1798083

[13] Tselios K, Sarantopoulos A, Gkougkourelas I, Boura P. The influence of therapy on CD4+CD25(high)FOXP3+ regulatory T cells in systemic lupus erythematosus patients: a prospective study. Scand J Rheumatol. 2015;44(1):29–35. Epub 2014/09/11. 10.3109/03009742.2014.922214 . 25205084

[14] Mosca M, Tani C, Carli L, Bombardieri S. Glucocorticoids in systemic lupus erythematosus. Clin Exp Rheumatol. 2011;29(5 Suppl 68):S126–9. Epub 2011/11/11. doi: 5433 [pii]. . 22018198

[15] Lisnevskaia L, Murphy G, Isenberg D. Systemic lupus erythematosus. Lancet. 2014;384(9957):1878–88. Epub 2014/06/03. 10.1016/S0140-6736(14)60128-8 S0140-6736(14)60128-8 [pii]. . 24881804

[16] Isenberg DA, Morrow WJ, Snaith ML. Methyl prednisolone pulse therapy in the treatment of systemic lupus erythematosus. Ann Rheum Dis. 1982;41(4):347–51. Epub 1982/08/01. 7114916 10.1136/ard.41.4.347 PMC1000949

[17] Mackworth-Young CG, David J, Morgan SH, Hughes GR. A double blind, placebo controlled trial of intravenous methylprednisolone in systemic lupus erythematosus. Ann Rheum Dis. 1988;47(6):496–502. Epub 1988/06/01. 3289511 10.1136/ard.47.6.496 PMC1003553

[18] Parker BJ, Bruce IN. High dose methylprednisolone therapy for the treatment of severe systemic lupus erythematosus. Lupus. 2007;16(6):387–93. Epub 2007/08/01. doi: 16/6/387 [pii] 10.1177/0961203307079502 . 17664228

[19] Badsha H, Edwards CJ. Intravenous pulses of methylprednisolone for systemic lupus erythematosus. Semin Arthritis Rheum. 2003;32(6):370–7. Epub 2003/07/02. 10.1053/sarh.2002.50003 S004901720270111X [pii]. . 12833245

[20] Hahn BH, McMahon MA, Wilkinson A, Wallace WD, Daikh DI, Fitzgerald JD, et al American College of Rheumatology guidelines for screening, treatment, and management of lupus nephritis. Arthritis Care Res (Hoboken). 2012;64(6):797–808. Epub 2012/05/05. 10.1002/acr.21664 22556106 PMC3437757

[21] Bertsias GK, Tektonidou M, Amoura Z, Aringer M, Bajema I, Berden JH, et al Joint European League Against Rheumatism and European Renal Association-European Dialysis and Transplant Association (EULAR/ERA-EDTA) recommendations for the management of adult and paediatric lupus nephritis. Ann Rheum Dis. 2012;71(11):1771–82. Epub 2012/08/02. doi: annrheumdis-2012-201940 [pii] 10.1136/annrheumdis-2012-201940 22851469 PMC3465859

[22] Ashwell JD, Lu FW, Vacchio MS. Glucocorticoids in T cell development and function. Annu Rev Immunol. 2000;18:309–45. Epub 2000/06/03. doi: 18/1/309 [pii] 10.1146/annurev.immunol.18.1.309 . 10837061

[23] Spies CM, Strehl C, van der Goes MC, Bijlsma JW, Buttgereit F. Glucocorticoids. Best Pract Res Clin Rheumatol. 2011;25(6):891–900. Epub 2012/01/24. 10.1016/j.berh.2011.11.002 S1521-6942(11)00153-7 [pii]. . 22265268

[24] Karagiannidis C, Akdis M, Holopainen P, Woolley NJ, Hense G, Ruckert B, et al Glucocorticoids upregulate FOXP3 expression and regulatory T cells in asthma. J Allergy Clin Immunol. 2004;114(6):1425–33. Epub 2004/12/04. doi: S009167490402010X [pii] 10.1016/j.jaci.2004.07.014 . 15577848

[25] Ling Y, Cao X, Yu Z, Ruan C. Circulating dendritic cells subsets and CD4+Foxp3+ regulatory T cells in adult patients with chronic ITP before and after treatment with high-dose dexamethasome. Eur J Haematol. 2007;79(4):310–6. Epub 2007/08/19. doi: EJH917 [pii] 10.1111/j.1600-0609.2007.00917.x . 17692100

[26] Xie Y, Wu M, Song R, Ma J, Shi Y, Qin W, et al A glucocorticoid amplifies IL-2-induced selective expansion of CD4(+)CD25(+)FOXP3(+) regulatory T cells in vivo and suppresses graft-versus-host disease after allogeneic lymphocyte transplantation. Acta Biochim Biophys Sin (Shanghai). 2009;41(9):781–91. Epub 2009/09/04. . 19727527 10.1093/abbs/gmp067

[27] Stary G, Klein I, Bauer W, Koszik F, Reininger B, Kohlhofer S, et al Glucocorticosteroids modify Langerhans cells to produce TGF-beta and expand regulatory T cells. J Immunol. 2011;186(1):103–12. Epub 2010/12/08. 10.4049/jimmunol.1002485 jimmunol.1002485 [pii]. . 21135170

[28] Bereshchenko O, Coppo M, Bruscoli S, Biagioli M, Cimino M, Frammartino T, et al GILZ promotes production of peripherally induced Treg cells and mediates the crosstalk between glucocorticoids and TGF-beta signaling. Cell Rep. 2014;7(2):464–75. Epub 2014/04/08. 10.1016/j.celrep.2014.03.004 S2211-1247(14)00166-1 [pii]. . 24703841

[29] Suarez A, Lopez P, Gomez J, Gutierrez C. Enrichment of CD4+ CD25high T cell population in patients with systemic lupus erythematosus treated with glucocorticoids. Ann Rheum Dis. 2006;65(11):1512–7. Epub 2006/04/12. doi: ard.2005.049924 [pii] 10.1136/ard.2005.049924 16606650 PMC1798359

[30] Cepika AM, Marinic I, Morovic-Vergles J, Soldo-Juresa D, Gagro A. Effect of steroids on the frequency of regulatory T cells and expression of FOXP3 in a patient with systemic lupus erythematosus: a two-year follow-up. Lupus. 2007;16(5):374–7. Epub 2007/06/20. doi: 16/5/374 [pii] 10.1177/0961203307077990 . 17576742

[31] Azab NA, Bassyouni IH, Emad Y, Abd El-Wahab GA, Hamdy G, Mashahit MA. CD4+CD25+ regulatory T cells (TREG) in systemic lupus erythematosus (SLE) patients: the possible influence of treatment with corticosteroids. Clin Immunol. 2008;127(2):151–7. Epub 2008/02/27. 10.1016/j.clim.2007.12.010 S1521-6616(08)00010-7 [pii]. . 18299252

[32] Hochberg MC. Updating the American College of Rheumatology revised criteria for the classification of systemic lupus erythematosus. Arthritis Rheum. 1997;40(9):1725. 10.1002/art.1780400928 9324032

[33] Bombardier C, Gladman DD, Urowitz MB, Caron D, Chang CH. Derivation of the SLEDAI. A disease activity index for lupus patients. The Committee on Prognosis Studies in SLE. Arthritis Rheum. 1992;35(6):630–40. 1599520 10.1002/art.1780350606

[34] Buyon JP, Petri MA, Kim MY, Kalunian KC, Grossman J, Hahn BH, et al The effect of combined estrogen and progesterone hormone replacement therapy on disease activity in systemic lupus erythematosus: a randomized trial. Ann Intern Med. 2005;142(12 Pt 1):953–62. 15968009 10.7326/0003-4819-142-12_part_1-200506210-00004

[35] Petri M, Kim MY, Kalunian KC, Grossman J, Hahn BH, Sammaritano LR, et al Combined oral contraceptives in women with systemic lupus erythematosus. N Engl J Med. 2005;353(24):2550–8. 16354891 10.1056/NEJMoa051135

[36] Miyara M, Chader D, Sage E, Sugiyama D, Nishikawa H, Bouvry D, et al Sialyl Lewis x (CD15s) identifies highly differentiated and most suppressive FOXP3high regulatory T cells in humans. Proc Natl Acad Sci U S A. 2015;112(23):7225–30. Epub 2015/05/28. 10.1073/pnas.1508224112 1508224112 [pii]. 26015572 PMC4466753

[37] Miyara M, Ito Y, Sakaguchi S. TREG-cell therapies for autoimmune rheumatic diseases. Nat Rev Rheumatol. 2014;10(9):543–51. Epub 2014/07/02. 10.1038/nrrheum.2014.105 nrrheum.2014.105 [pii]. . 24980140

[38] Prado C, Gomez J, Lopez P, de Paz B, Gutierrez C, Suarez A. Dexamethasone upregulates FOXP3 expression without increasing regulatory activity. Immunobiology. 2011;216(3):386–92. Epub 2010/07/30. 10.1016/j.imbio.2010.06.013 S0171-2985(10)00120-8 [pii]. . 20667622

[39] Sbiera S, Dexneit T, Reichardt SD, Michel KD, van den Brandt J, Schmull S, et al Influence of short-term glucocorticoid therapy on regulatory T cells in vivo. PLoS One. 2011;6(9):e24345 Epub 2011/09/14. 10.1371/journal.pone.0024345 PONE-D-11-07764 [pii]. 21912688 PMC3166315

[40] Seissler N, Schmitt E, Hug F, Sommerer C, Zeier M, Schaier M, et al Methylprednisolone treatment increases the proportion of the highly suppressive HLA-DR(+)-Treg-cells in transplanted patients. Transpl Immunol. 2012;27(4):157–61. Epub 2012/10/02. 10.1016/j.trim.2012.09.003 S0966-3274(12)00094-9 [pii]. . 23022208

[41] Miroux C, Morales O, Carpentier A, Dharancy S, Conti F, Boleslowski E, et al Inhibitory effects of cyclosporine on human regulatory T cells in vitro. Transplant Proc. 2009;41(8):3371–4. Epub 2009/10/28. 10.1016/j.transproceed.2009.08.043 S0041-1345(09)01236-6 [pii]. . 19857752

[42] van de Wetering J, Koumoutsakos P, Peeters A, van der Mast BJ, de Kuiper P, JN IJ, et al Discontinuation of calcineurin inhibitors treatment allows the development of FOXP3+ regulatory T-cells in patients after kidney transplantation. Clin Transplant. 2011;25(1):40–6. Epub 2010/07/20. 10.1111/j.1399-0012.2010.01311.x CTR1311 [pii]. . 20636406

[43] Chen X, Murakami T, Oppenheim JJ, Howard OM. Differential response of murine CD4+CD25+ and CD4+CD25- T cells to dexamethasone-induced cell death. Eur J Immunol. 2004;34(3):859–69. Epub 2004/03/03. 10.1002/eji.200324506 . 14991616

[44] Nicolaides NC, Galata Z, Kino T, Chrousos GP, Charmandari E. The human glucocorticoid receptor: molecular basis of biologic function. Steroids. 2010;75(1):1–12. Epub 2009/10/13. 10.1016/j.steroids.2009.09.002 S0039-128X(09)00208-6 [pii]. 19818358 PMC2813911

[45] Rudra D, deRoos P, Chaudhry A, Niec RE, Arvey A, Samstein RM, et al Transcription factor Foxp3 and its protein partners form a complex regulatory network. Nat Immunol. 2012;13(10):1010–9. Epub 2012/08/28. 10.1038/ni.2402 ni.2402 [pii]. 22922362 PMC3448012

[46] Hasegawa H, Inoue A, Muraoka M, Yamanouchi J, Miyazaki T, Yasukawa M. Therapy for pneumonitis and sialadenitis by accumulation of CCR2-expressing CD4+CD25+ regulatory T cells in MRL/lpr mice. Arthritis Res Ther. 2007;9(1):R15. Epub 2007/02/08. doi: ar2122 [pii] 10.1186/ar2122 17284325 PMC1860074

[47] Weigert O, von Spee C, Undeutsch R, Kloke L, Humrich JY, Riemekasten G. CD4+Foxp3+ regulatory T cells prolong drug-induced disease remission in (NZBxNZW) F1 lupus mice. Arthritis Res Ther. 2013;15(1):R35 Epub 2013/03/01. 10.1186/ar4188 ar4188 [pii]. 23446139 PMC3672693

[48] Moniuszko M, Bodzenta-Lukaszyk A, Dabrowska M. Effects of oral glucocorticoid therapy on CD4+CD25+CD127- and CD4+CD25high T cell levels in asthmatic patients. Inflammation. 2010;33(6):415–20. Epub 2010/03/20. 10.1007/s10753-010-9200-9 . 20300815

[49] Ballou SP, Khan MA, Kushner I. Intravenous pulse methylprednisolone followed by alternate day corticosteroid therapy in lupus erythematosus: a prospective evaluation. J Rheumatol. 1985;12(5):944–8. Epub 1985/10/01. . 3910835

[50] Noel V, Lortholary O, Casassus P, Cohen P, Genereau T, Andre MH, et al Risk factors and prognostic influence of infection in a single cohort of 87 adults with systemic lupus erythematosus. Ann Rheum Dis. 2001;60(12):1141–4. Epub 2001/11/16. 11709457 10.1136/ard.60.12.1141 PMC1753456

[51] Saadoun D, Rosenzwajg M, Joly F, Six A, Carrat F, Thibault V, et al Regulatory T-cell responses to low-dose interleukin-2 in HCV-induced vasculitis. N Engl J Med. 2011;365(22):2067–77. Epub 2011/12/02. 10.1056/NEJMoa1105143 . 22129253

[52] Koreth J, Matsuoka K, Kim HT, McDonough SM, Bindra B, Alyea EP 3rd, et al Interleukin-2 and regulatory T cells in graft-versus-host disease. N Engl J Med. 2011;365(22):2055–66. Epub 2011/12/02. 10.1056/NEJMoa1108188 22129252 PMC3727432

[53] Long SA, Rieck M, Sanda S, Bollyky JB, Samuels PL, Goland R, et al Rapamycin/IL-2 combination therapy in patients with type 1 diabetes augments Tregs yet transiently impairs beta-cell function. Diabetes. 2012;61(9):2340–8. Epub 2012/06/23. 10.2337/db12-0049 db12-0049 [pii]. 22721971 PMC3425404

[54] Humrich JY, Morbach H, Undeutsch R, Enghard P, Rosenberger S, Weigert O, et al Homeostatic imbalance of regulatory and effector T cells due to IL-2 deprivation amplifies murine lupus. Proc Natl Acad Sci U S A. 2010;107(1):204–9. Epub 2009/12/19. 10.1073/pnas.0903158107 0903158107 [pii]. 20018660 PMC2806746

[55] Mizui M, Koga T, Lieberman LA, Beltran J, Yoshida N, Johnson MC, et al IL-2 protects lupus-prone mice from multiple end-organ damage by limiting CD4-CD8- IL-17-producing T cells. J Immunol. 2014;193(5):2168–77. Epub 2014/07/27. 10.4049/jimmunol.1400977 jimmunol.1400977 [pii]. 25063876 PMC4135016

[56] von Spee-Mayer C, Siegert E, Abdirama D, Rose A, Klaus A, Alexander T, et al Low-dose interleukin-2 selectively corrects regulatory T cell defects in patients with systemic lupus erythematosus. Ann Rheum Dis. 2015. Epub 2015/09/02. doi: annrheumdis-2015-207776 [pii] 10.1136/annrheumdis-2015-207776 . 26324847

[57] Humrich JY, von Spee-Mayer C, Siegert E, Alexander T, Hiepe F, Radbruch A, et al Rapid induction of clinical remission by low-dose interleukin-2 in a patient with refractory SLE. Ann Rheum Dis. 2015;74(4):791–2. Epub 2015/01/23. 10.1136/annrheumdis-2014-206506 annrheumdis-2014-206506 [pii]. . 25609413

[58] Chen X, Oppenheim JJ, Winkler-Pickett RT, Ortaldo JR, Howard OM. Glucocorticoid amplifies IL-2-dependent expansion of functional FoxP3(+)CD4(+)CD25(+) T regulatory cells in vivo and enhances their capacity to suppress EAE. Eur J Immunol. 2006;36(8):2139–49. Epub 2006/07/15. 10.1002/eji.200635873 . 16841298

[59] Seddiki N, Santner-Nanan B, Martinson J, Zaunders J, Sasson S, Landay A, et al Expression of interleukin (IL)-2 and IL-7 receptors discriminates between human regulatory and activated T cells. J Exp Med. 2006;203(7):1693–700. Epub 2006/07/05. doi: jem.20060468 [pii] 10.1084/jem.20060468 16818676 PMC2118333

[60] Fontenot JD, Rasmussen JP, Williams LM, Dooley JL, Farr AG, Rudensky AY. Regulatory T cell lineage specification by the forkhead transcription factor foxp3. Immunity. 2005;22(3):329–41. Epub 2005/03/23. doi: S1074-7613(05)00066-X [pii] 10.1016/j.immuni.2005.01.016 . 15780990

[61] Sugimoto N, Oida T, Hirota K, Nakamura K, Nomura T, Uchiyama T, et al Foxp3-dependent and -independent molecules specific for CD25+CD4+ natural regulatory T cells revealed by DNA microarray analysis. Int Immunol. 2006;18(8):1197–209. Epub 2006/06/15. doi: dxl060 [pii] 10.1093/intimm/dxl060 . 16772372

[62] Venigalla RK, Tretter T, Krienke S, Max R, Eckstein V, Blank N, et al Reduced CD4+,CD25- T cell sensitivity to the suppressive function of CD4+,CD25high,CD127 -/low regulatory T cells in patients with active systemic lupus erythematosus. Arthritis Rheum. 2008;58(7):2120–30. Epub 2008/06/26. 10.1002/art.23556 . 18576316

[63] Bonelli M, Savitskaya A, Steiner CW, Rath E, Smolen JS, Scheinecker C. Phenotypic and functional analysis of CD4+ CD25- Foxp3+ T cells in patients with systemic lupus erythematosus. J Immunol. 2009;182(3):1689–95. Epub 2009/01/22. doi: 182/3/1689 [pii]. . 19155519 10.4049/jimmunol.182.3.1689

[64] Alexander T, Sattler A, Templin L, Kohler S, Gross C, Meisel A, et al Foxp3+ Helios+ regulatory T cells are expanded in active systemic lupus erythematosus. Ann Rheum Dis. 2013;72(9):1549–58. Epub 2012/12/25. 10.1136/annrheumdis-2012-202216 annrheumdis-2012-202216 [pii]. . 23264341

[65] Golding A, Hasni S, Illei G, Shevach EM. The percentage of FoxP3+Helios+ Treg cells correlates positively with disease activity in systemic lupus erythematosus. Arthritis Rheum. 2013;65(11):2898–906. Epub 2013/08/09. 10.1002/art.38119 23925905 PMC3891045

[66] Joller N, Lozano E, Burkett PR, Patel B, Xiao S, Zhu C, et al Treg cells expressing the coinhibitory molecule TIGIT selectively inhibit proinflammatory Th1 and Th17 cell responses. Immunity. 2014;40(4):569–81. Epub 2014/04/22. 10.1016/j.immuni.2014.02.012 S1074-7613(14)00106-X [pii]. 24745333 PMC4070748

[67] Bin Dhuban K, d'Hennezel E, Nashi E, Bar-Or A, Rieder S, Shevach EM, et al Coexpression of TIGIT and FCRL3 identifies Helios+ human memory regulatory T cells. J Immunol. 2015;194(8):3687–96. Epub 2015/03/13. 10.4049/jimmunol.1401803 jimmunol.1401803 [pii]. . 25762785 PMC4610024

