# Supplementary figures and images for: Regulatory T Cell Responses to High-Dose Methylprednisolone in Active Systemic Lupus Erythematosus
Source: PLoS One. 2015 Dec 2;10(12):e0143689. doi: 10.1371/journal.pone.0143689 (PMC4667921; doi:10.1371/journal.pone.0143689)

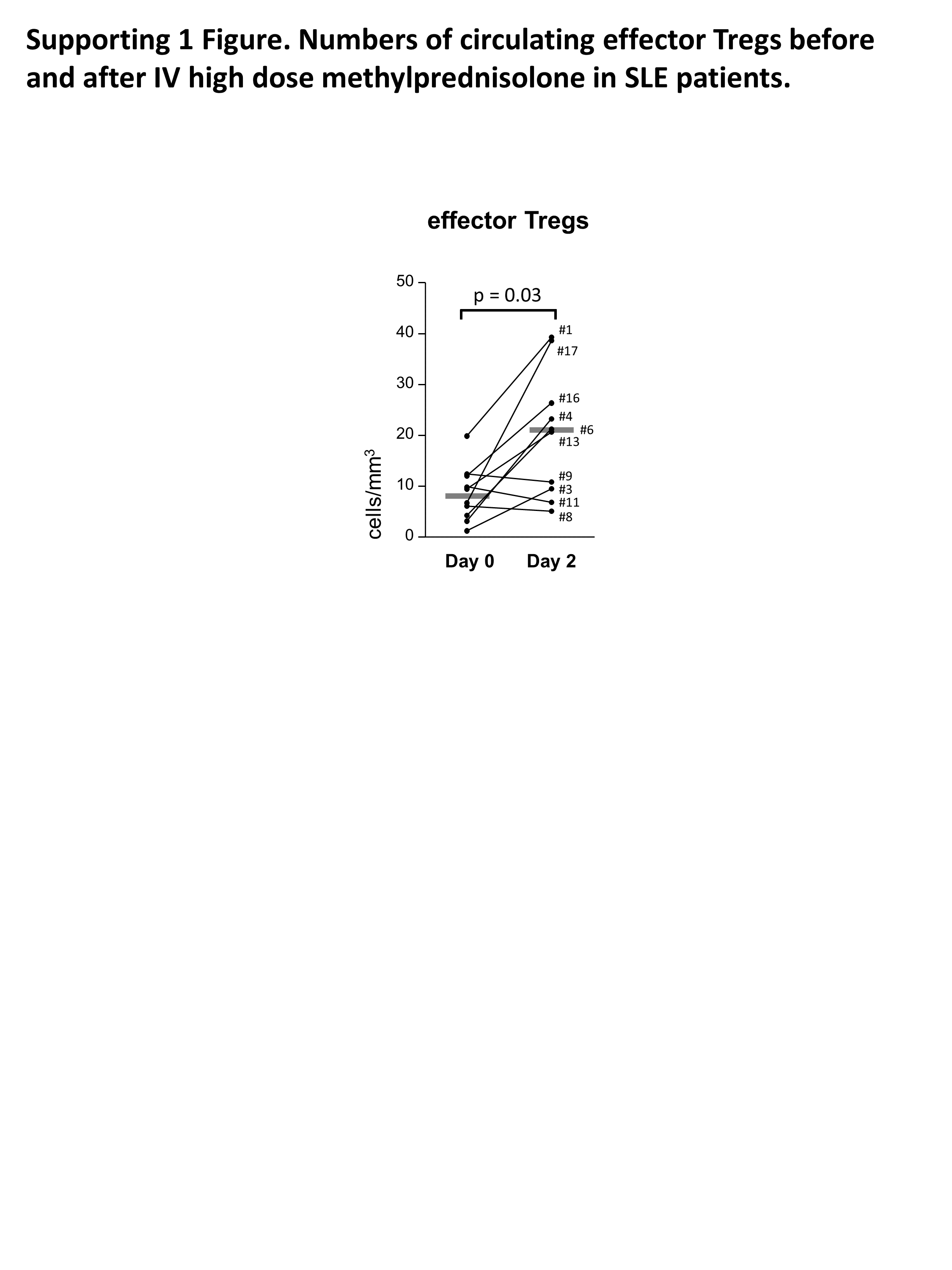

Supplement: S1 Fig — The frequency of eTreg were determined by flow cytofluorometry as shown in Fig 1. The absolute counts for circulating eTreg cells were calculated by multiplying the frequencies of eTreg cells among CD4+ T cells with the frequencies of CD4+ T cells among total lymphocytes and the total lymphocyte blood count which was available at day 3 only for 10 patients. Each dot represents an individual assessed in an independent experiment, and the grey bar shows median values. Statistical analyses were performed using the Wilcoxon matched pairs signed ranks test. (TIF) [file pone.0143689.s001.tif]

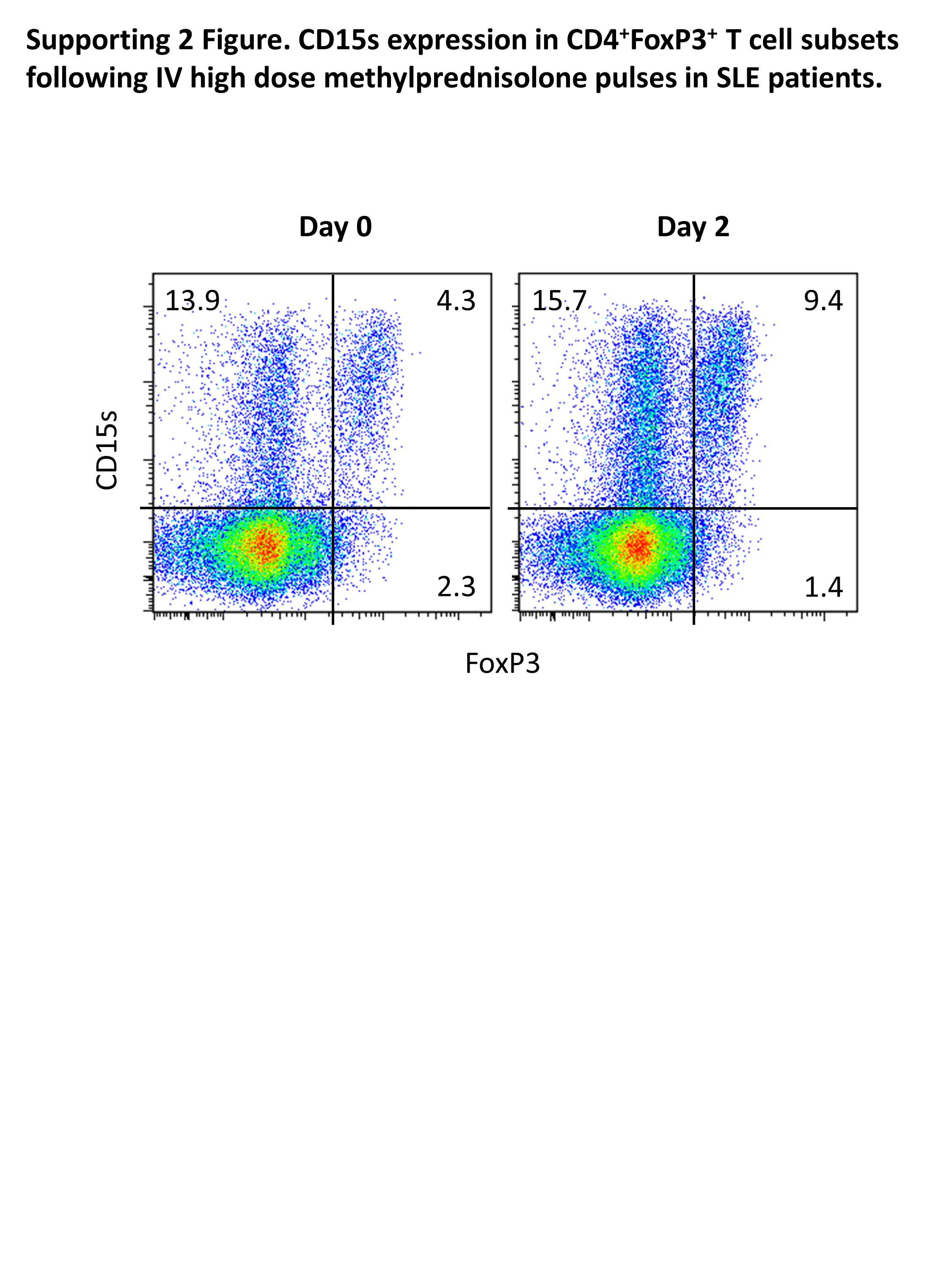

Supplement: S2 Fig — Fresh PBMCs from SLE patients were analyzed by flow cytofluorometry, gated on CD4+ T lymphocytes, for the expression of FoxP3 and CD15s before IV high dose MP pulses i.e., day 0 and at day 2 after the first pulse. Percentages of the different subsets are shown. Representative analyses from one SLE patient are shown (pt #15). (TIF) [file pone.0143689.s002.tif]
